# Decreased microglial activation through gut-brain axis by prebiotics, probiotics, or synbiotics effectively restored cognitive function in obese-insulin resistant rats

**DOI:** 10.1186/s12974-018-1055-2

**Published:** 2018-01-09

**Authors:** Titikorn Chunchai, Wannipa Thunapong, Sakawdaurn Yasom, Keerati Wanchai, Sathima Eaimworawuthikul, Gabrielle Metzler, Anusorn Lungkaphin, Anchalee Pongchaidecha, Sasithorn Sirilun, Chaiyavat Chaiyasut, Wasana Pratchayasakul, Parameth Thiennimitr, Nipon Chattipakorn, Siriporn C. Chattipakorn

**Affiliations:** 1 Neurophysiology Unit, Cardiac Electrophysiology Research and Training Center, Faculty of Medicine, Chiang Mai University, Chiang Mai, 50200 Thailand; 2 Cardiac Electrophysiology Research and Training Center, Department of Physiology, Faculty of Medicine, Chiang Mai University, Chiang Mai, 50200 Thailand; 3 Department of Microbiology, Faculty of Medicine, Chiang Mai University, Chiang Mai, 50200 Thailand; 4 Faculty of Pharmacy, Chiang Mai University, Chiang Mai, 50200 Thailand; 5 Department of Oral Biology and Diagnostic Science, Faculty of Dentistry, Chiang Mai University, Chiang Mai, 50200 Thailand

**Keywords:** Xyloolidosaccharide, *Lactobacillus paracasei* HII01, Synbiotics, Microglia, Brain mitochondrial function, Cognitive function

## Abstract

**Background:**

Chronic high-fat diet (HFD) consumption caused not only obese-insulin resistance, but also cognitive decline and microglial hyperactivity. Modified gut microbiota by prebiotics and probiotics improved obese-insulin resistance. However, the effects of prebiotics, probiotics, and synbiotics on cognition and microglial activity in an obese-insulin resistant condition have not yet been investigated. We aimed to evaluate the effect of prebiotic (Xyloolidosaccharide), probiotic (*Lactobacillus paracasei* HII01), or synbiotics in male obese-insulin resistant rats induced by a HFD.

**Methods:**

Male Wistar rats were fed with either a normal diet or a HFD for 12 weeks. At week 13, the rats in each dietary group were randomly divided into four subgroups including vehicle group, prebiotics group, probiotics group, and synbiotics group. Rats received their assigned intervention for an additional 12 weeks. At the end of experimental protocol, the cognitive functioning of each rat was investigated; blood and brain samples were collected to determine metabolic parameters and investigate brain pathology.

**Results:**

We found that chronic HFD consumption leads to gut and systemic inflammation and impaired peripheral insulin sensitivity, which were improved by all treatments. Prebiotics, probiotics, or synbiotics also improved hippocampal plasticity and attenuated brain mitochondrial dysfunction in HFD-fed rats. Interestingly, hippocampal oxidative stress and apoptosis were significantly decreased in HFD-fed rats with all therapies, which also decreased microglial activation, leading to restored cognitive function.

**Conclusions:**

These findings suggest that consumption of prebiotics, probiotics, and synbiotics restored cognition in obese-insulin resistant subjects through gut-brain axis, leading to improved hippocampal plasticity, brain mitochondrial function, and decreased microglial activation.

## Background

Obesity has reached epidemic proportions in many countries around the world [1]. Obesity is also known to lead to the development of insulin resistance [2, 3] and is associated with learning impairment and memory decline [4]. Growing evidence from our group have clearly demonstrated that obesity in rats, induced by long-term high-fat diet (HFD) consumption, not only caused peripheral insulin resistance, but also brain insulin resistance, dyslipidemia, and increased oxidative stress [3]. Furthermore, chronic HFD-fed rats have been shown to have the hippocampal synaptic dysfunction as indicated by the impairment of long-term potentiation (LTP) and dendritic spine loss, leading to cognitive decline [4–6]. In addition, mitochondrial dysfunction has been associated with a cognitive decline in rats fed chronically with HFD [5, 7–14].

Recently, the role of gut microbiota, a group of beneficial microbes living inside the gastrointestinal tract, has been revealed in several human diseases including obesity [15]. Human and rodents shared the similarity of gut microbiota in the phylum level which composed of the five major phyla including *Firmicutes*, *Bacteroidetes*, *Actinobacteria*, *Proteobacteria*, and *Verrucomicrobia* [16, 17]. Prolonged consumption of HFD resulted in an imbalance of gut microbiota termed “gut dysbiosis” by increasing the ratio of *Firmicutes* to *Bacteroidetes* (F/B ratio) and promoting the growth of *Proteobacteria* [18, 19]. Cumulative evidence showed that the modulation of gut microbiota by prebiotics and probiotics could be effective therapeutic strategies to improve obesity and insulin resistance [20]. Prebiotics, non-digestible food ingredients which were digested by gut microbiota [21], and probiotics, live micro-organisms which, when administered in adequate amount, confer health benefits on the host [22], showed favorable effects by altering the composition and metabolism of gut microbiota and improved metabolic function in various animal models of metabolic syndrome [23]. Previous study also demonstrated that consumption of probiotics had beneficial effects to the brain through gut-brain axis [24]. Although probiotics had been shown to improve cognition and anxiety in hyperammonemia rats and also attenuated depression in humans [25], inconsistent reports exist in which probiotics failed to modulate stress or cognitive performance in healthy male subjects [26]. Furthermore, recent studies demonstrated that consumption of 10% of probiotic xylooligosaccharide (XOS) reduced the body weight, blood glucose, and cholesterol in streptozotozin-induced diabetic rats [27]. In addition, a previous study demonstrated that 10^8^ colony-forming unit (cfu) of the *Lactobacillus paracasei* HII01 could survive in the acidic environment of the gastrointestinal tract and in the presence of gastric enzymes, bile salts, and considered as a safe dose [28].

Microglia, the brain resident macrophage, has been proposed to play a crucial role in neurodegenerative disorders. It has been shown that microglia excessively pruned synapses and increased pro-inflammatory cytokines in models of Alzheimer’s disease [29, 30]. Microglia are also associated with cognitive function [31]. Chronic HFD consumption has been shown to trigger microglial activation, leading to cognitive impairment [31–33]. Recent studies also illustrated the communication linking between microglial function and host microbiota [34, 35]. Moreover, it has been shown that gut microbiota could modulate key transcriptional co-activators, transcription factors, and enzymes involved in mitochondrial biogenesis [36]. Since mitochondria are the major producer of reactive oxygen species (ROS) [37], which could cause microglia activation [38, 39], these ROS and pro-inflammatory cytokines released from activated microglia inhibited LTP, resulting in cognitive impairment [40, 41]. In addition, pro-inflammatory cytokines could also activate intrinsic apoptotic pathway [42], which was attenuated by prebiotic and probiotics therapy [43].

Despite these previous findings, the effects of prebiotic XOS, probiotic *L. paracasei* HII01, or its combination, an equal amount of XOS and *L. paracasei* HII01 as a synbiotics, on the modulation of microglia and cognitive functions by altering gut microbiota composition in an obese-insulin resistant model have not been investigated. We tested the hypothesis that prebiotic, probiotic, or synbiotics in obese-insulin resistant rats induced by chronic HFD consumption reduces gut dysbiosis and improves cognitive function by attenuating gut inflammation, peripheral insulin resistance, restoring hippocampal synaptic plasticity, decreasing brain mitochondrial dysfunction and hippocampal oxidative stress and apoptosis, and preserving microglial morphology.

## Methods

### Animals and diet

All animal studies were approved by the Institutional Animal Care and Use Committee (IACUC) of the Faculty of Medicine, Chiang Mai University (Permit number: 13/2558 on May 12, 2015) and conformed to the Guide for the Care and Use of Laboratory Animals published by the US National Institutes of Health (NIH guide, 8th edition, 2011). Male Wistar rats (180–200 g) were purchased from the National Laboratory Animal Center, Salaya campus, Mahidol University, Bangkok, Thailand. All rats were housed individually in a temperature-controlled environment (25 ± 0.5 °C) with a 12:12 h light-dark cycle. After 1 week of acclimatization, animals were fed with either a normal diet (ND; 19.77% energy from fat) or a high-fat diet (HFD; 59.28% energy from fat) for 12 weeks. All rats received reverse osmosis drinking water ad libitum. Food intake was recorded daily and body weight was recorded weekly. After 12 weeks, blood collection and behavioral assessment were measured in all animals. At week 13, the rats in each dietary group were randomly divided into four subgroups including ND- and HFD-fed rats oral feeding with phosphate buffer saline (PBS) as the vehicle group (NDV and HFV); ND- and HFD-fed rats oral feeding with prebiotics (10% XOS in PBS, 1 ml/day; NDPE and HFPE); ND- and HFD-fed rats oral feeding with probiotics (1 × 10^8^ cfu of *L. paracasei* HII01, 1 ml/day; NDPO and HFPO), and ND- and HFD-fed rats oral feeding with 2 ml of synbiotics (a 1:1 mixture of 10% XOS and 10^8^ cfu *L. paracasei* HII01; NDC and HFC). For prebiotics, 10% of XOS has been shown to reduce the body weight, blood glucose, and cholesterol in streptozotozin-induced diabetic rats [27]. For probiotics, a previous study demonstrated that 10^8^ CFU of the *L. paracasei* HII01, which is a live microorganism, could survive in the acidic environment of the gastrointestinal tract and in the presence of gastric enzymes, bile salts, and considered as a safety dose [28]. The prebiotic XOS was purchased from Shandong Longlive Biotechnology CO., LTD., Shandong, China, and probiotic *L. paracasei* HII01 was kindly provided by the Department of Pharmaceutical Sciences, Faculty of Pharmacy, Chiang Mai University, Thailand. Rats received their assigned intervention for an additional 12 weeks.

**Fig. 1:**
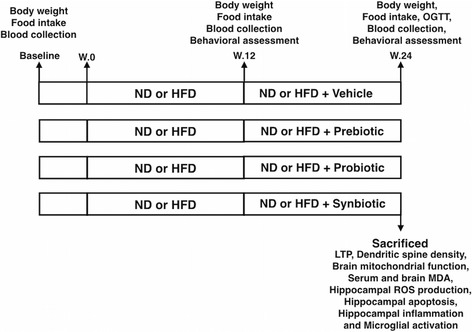
The experimental protocol of the present study

At the end of the experimental protocol, the cognitive functioning of each rat was investigated and the oral glucose tolerance test (OGTT) was performed. Then, rats (*n* = 6/subgroup) were deeply anesthetized with isoflurane and killed by decapitation. The brain of each rat was quickly removed and carefully sliced in preparation for investigation, including extracellular recording (electrical-induced LTP) for hippocampal plasticity, brain mitochondrial function, hippocampal ROS production, and hippocampal apoptosis. Another group of rats (*n* = 6/subgroup) was also deeply anesthetized with isoflurane and subsequently perfused with 4% paraformaldehyde for determining microglial morphology. The experimental protocol is summarized in Fig. 1.

### Metabolic parameters determination

Plasma glucose and cholesterol levels were determined via colorimetric assay (Biotech, Bangkok, Thailand). The commercial colorimetric assay kit (Biovision, CA, USA) was used for determining plasma total LDL levels. Plasma insulin levels were also determined using the Sandwich ELISA kit (LINCO Research, MO, USA). Homeostasis Model Assessment (HOMA) was used for assessing the peripheral insulin resistance as described in previous studies [44, 45]. OGTT was performed as described by Pintana et al. [5]. Briefly, rats were fasted overnight before the test and received 2 g/kg of glucose solution via oral gavage feeding. Blood samples were collected from the tail vein at 0, 15, 30, 60, 90, and 120 min after glucose administration. Areas under the curve (AUC) were calculated to evaluate glucose tolerance. To examine the brain oxidative stress, hippocampal malondialdehyde (MDA) level was determined by high-performance liquid chromatography (HPLC), as described in the previous studies [46]. Serum lipopolysaccharide (LPS) levels were measured by colorimetric method using the Pierce® LAL Chromogenic Endotoxin Quantitation Kit (Thermo Fisher Scientific, USA). Serum was diluted (1:10) with sterile endotoxin-free water and inactivated at 70 °C for 15 min. Then, the heat-inactivated serum was incubated with limulus amoebocyte lysate (LAL) at 37° for 10 min as described previously [19]. Next, substrate solution was added; the development of magenta-colored derivative was detected using the absorbance at 410 nm. The concentrations of serum LPS in the samples were then calculated using the standard curve and reported in EU/mL.

### Tissue and brain slice preparation

Brain tissue in each rat was removed and immersed in ice-cold artificial cerebrospinal fluid (aCSF) containing high sucrose for 30 min. Brain slices (400 μm) were cut on a vibratome (Vibratome Company, MO, USA). The slices were transferred to a room temperature (22–24 °C) CSF solution for an additional 30 min and subsequently transferred to a recording chamber containing standard aCSF for extracellular recording. Other brain tissue or hippocampi were homogenated in solution buffer containing protease inhibiter for brain mitochondrial function, hippocampal ROS production, and immunoblotting.

### Quantitative real-time PCR analysis

Transcription levels of pro-inflammatory cytokine genes, IL-1β (5*′*-CACCTCTCAAGCAGAGCACAG-3*′* and 5*′*-GGGTTCCATGGTGAAGTCAAC-3′), IL-6 (5*′*-TCCTACCCCAACTTCCAATGCTC-3′ and 5*′*-TTGGATGGTCTTGGTCCTTAGCC-3′), and immunosuppressive cytokine IL-10 (5*′*-AGTCAGCCAGACCCACAT-3′ and 5*′*-GGCAACCCAAGTAACCCT-3′) were determined as previously described [47]. In brief, the frozen colon and brain tissues in RNA preservative solution were homogenized by using 1 mm sterile zirconia/silica bead (Biospec Products, Bartlesville, US) and Minibeadbeater (Biospec Products, Bartlesville, US). Next, homogenized tissues were extracted from RNA using TRI reagent (TRIzol® Reagent, Ambion, Life Technologies, CA, US) according to the recommendations of the manufacturer. Then, a DNase treatment was performed by adding the DNA removal and inactivation kit (Ambion, Life Technologies, CA, US). The extracted tissue RNA was converted to complementary DNA (cDNA) using reverse transcription reagents (Tetro cDNA synthesis kit, Bioline, US). SYBR-Green (SensiFAST SYBR Lo-ROX kit, Bioline, US)-based real-time quantitative PCR was conducted using the primers and further analyzed by comparative Ct method. The mRNA expression levels of target genes were normalized with Gapdh (5′-GTATTGGGCGCCTGGTCACC-3′ and 5′*-*CGCTCCTGGAAGATGGTGATGG-3′) mRNA levels.

### Extracellular recordings of hippocampal slices long-term potentiation

To determine hippocampal plasticity, the field excitatory postsynaptic potentials (fEPSP) slope of LTP was measured from CA1 area of hippocampal slices. LTP is a marker of hippocampal synaptic plasticity. [3]. Briefly, brain slices were transferred to a submersion recording chamber and continuously perfused at 3–4 ml/min with standard aCSF warmed to 28–29 °C. Field excitatory postsynaptic potentials (fEPSPs) were evoked by stimulating the Schaffer collateral-commissural pathway with a bipolar tungsten electrode, while the fEPSPs recordings were taken from the stratum radiatum of the hippocampal CA1 region with micropipettes (3 MW) filled with 2M NaCl. LTP was induced by delivering high-frequency tetani [high-frequency stimulation (HFS); four trains at 100 Hz; 0.5 s duration; 20 s interval] at 1.5 times the baseline stimulation intensity. Experiments were performed for at least 40 min after HFS. The amount of potentiation was calculated at 40 min after tetanus. Data were filtered at 3 kHz, digitized at 10 kHz, and stored in a computer using pClamp9.2 software (Axon Instruments, CA, USA). The initial slope of the fEPSPs was measured and plotted against time [3, 8, 9, 11–14, 48].

### Brain mitochondrial function

Brain mitochondria were isolated as described in Pipatpiboon et al. [11]. Mitochondrial protein was determined by the BCA assay as described previously [5], and brain mitochondrial function including brain mitochondrial ROS, mitochondrial membrane potential change (ΔΨm), and mitochondrial swelling was determined [13, 14, 48]. Brain mitochondrial ROS were measured using dichloro-hydrofluoresceindiacetate (DCFHDA) fluorescent dye. The change in mitochondrial membrane potential (ΔΨm) was measured using the fluorescent dye 5, 5¢, 6, 6¢-tetrachloro-1, 1¢, 3, 3¢-tetraethyl benzimidazolcarbocyanine iodide (JC-1), and brain mitochondrial swelling was determined by measuring the change in the absorbance of brain mitochondrial suspension at 540 nm. All were determined by following the methods described previously [13, 14, 48].

### Immunoblotting of hippocampal apoptotic and anti-apoptotic proteins

To investigate the hippocampal apoptosis, homogenate hippocampi were used, as described in the references [13, 14, 48]. Examination of the level of apoptotic and anti-apoptotic protein expression was conducted with homogenates prepared from hippocampus tissue. These proteins were separated and identified by an immunoblot assay conducted with rabbit anti-bax (1:200; Santa Cruz Biotechnology, CA, USA), bcl-2 (1:1000; Cell Signaling Technology, MA, USA). For a loading control, immunoblotting for each membrane was incubated with anti-β-actin (1:4000; #4967; Cell Signaling Technology, MA, USA). All membranes were incubated with a secondary goat anti-rabbit antibody conjugated with horseradish peroxidase (1:2000; #7074; Cell Signaling Technology, MA, USA). The protein bands were visualized on ChemiDocÔ touch imaging system (Bio-Rad, CA, USA) using Amersham ECL Western blot detection reagents (GE Healthcare, Buckinghamshire, UK). The band intensity was measured by Scion Image, and the results were represented as average signal intensity (arbitrary) units.

#### Immunofluorescent labeling for hippocampal plasticity, microglial morphology, and image analysis

Animals were transcardially perfused with 4% paraformaldehyde, postfix for an additional 24 h, cryoprotected in 30% sucrose in PBS at 4 °C, and then frozen in isopentane and dry ice, and stored at − 80 °C. Then, the brains were cut using cryosection (Leica CM1950, Leica Biosystem Nussloch GmbH, Nussloch, Germany) at 20 μm. Sections were subjected to label immunofluorescence. The sections were quenched with 3% peroxide, blocked with 5% BSA, and incubated overnight at 4 °C with primary antibodies for Iba-1 (ab5076, Abcam, Cambridge, MA) for microglia morphology [32]. After being washed three times in TBS, sections were incubated with AlexaFluor conjugated secondary antibodies; Iba1- AlexaFluor 488 anti-goat, for 1 h at 25 °C then rinsed in TBS. Sections were treated with copper sulfate in ammonium acetate buffer to quench endogenous autofluorescence of the brain tissue. To determine the microglial morphology, the series of z-stacks of microglia images were taken from confocal microscopy (Olympus flouview FV3000) and microglial morphology was measured by Imaris software 7.0 (Bitplane, Oxford instrument company, AG, Zurich, Switzerland). Three microglial cells per brain slice, three brain slices per animal and six animals per group were measured from the CA1 region of the hippocampus. All microglial morphology parameters including soma area, processes length and the number of primary branch projection (ramification) were measured from a 3D constructuring using Imaris. The number of Iba-1 positive cells and the mean fluorescent intensity were also measured. For visualization of dendritic spines, slices were labeled with the carbocyanine dye 1,1′-dioctadecyl-3,3,3′,3’-Tetramethylindocarbocyanine Perchlorate (DiI; Invitrogen), as described previously [32, 33]. Slices were incubated with appropriately placed DiI crystals for 48–72 h before being mounted on slides and coverslipped in 0.1 M Tris buffer. Sections were mounted on slides and coverslipped by the anti-fading mounting medium Fluoromount (Sigma-Aldrich Chemie, Steinheim, Germany). To assess the dendritic spine density, a series of 10 optical sections were taken every 0.25 mm in the *z*-plane, stacked into z-stacks of 2.5 mm, and shown as a z-projection of the total z-stack. For spine analysis, the three tertiary segments, 100–200 μm apart from the soma and 20–30 μm in dendritic length, were used to randomly measure dendritic spine density. Three neuronal cells per brain slice and three brain slices per animal were chosen for spine quantitative analysis. The number of spines was counted by double-blind hand counter [48].

### Cognitive function test

The Morris water maze test was performed to determine cognitive function with two assessments, including five consecutive days of the acquisition test, and the probe test on day sixth. Time to find the platform was recorded in the acquisition test, and the time spent in the target quadrant was also recorded in the probe test [46, 49]. Data analysis of the MWM test was done manually from videotape recordings by the investigators, who were blinded to experimental groups. To determine locomotor activity, all animals were tested by open-field test [50, 51]. In this method, the apparatus consists of a rectangular-based box open from above (70 cm long and wide, and 90 cm in height). Each animal was placed into the box and allowed for 5-min exploration. After 10 mins of exploration time, the animals were taken out. The distance was counted using SMART 3.0 software (Panlab®, Harvard Apparatus, Barcelona, Spain).

#### Gut microbiota analysis

Feces of each animal were collected at the end of experimental protocol. Bacterial genomic DNA was extracted from rat fecal pellet using a commercial genomic DNA isolation kit (QIAGEN, Germany). Briefly, the fecal sample (0.25 g) was homogenized in QIAGEN ASL lysis buffer by a Minibeadbeater (BioSpec products, Bartlesville, USA). The extraction of bacterial genomic DNA was done following the manufacturer’s instruction. The fractions of bacterial microbiota population (*Firmicutes/Bacteroidetes* ratio) were quantified using real-time quantitative reverse transcription PCR (qRT-PCR) as described previously [52].

#### Statistical analysis

Data from each experiment were expressed as mean ± S.E.M. For all multiple comparisons, data were analyzed using a two-way ANOVA, followed by post-hoc Tukey’s analysis. Correlations and regression analysis were also conducted to look at relationships between metabolic parameters and behavioral test. For behavioral test, the significance of the difference of acquisition test was calculated using repeated two-way ANOVA, followed by post-hoc Tukey’s analysis. The significance of the difference of probe test at week 12 was calculated using an independent *t* test. A *p* < 0.05 was considered as statistically significant.

## Results

### Long-term HFD consumption induced gut dysbiosis and systemic inflammation, which was attenuated by prebiotic XOS, probiotic *L. paracasei* HIIO1, or synbiotics

**Fig. 2:**
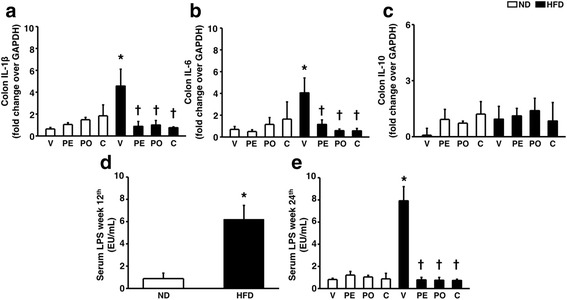
Effects of prebiotics, probiotics, or synbiotics on gut inflammation and endotoxemia induced by long-term HFD consumption. **a**–**c** The pro-inflammatory cytokine including IL-1β expression, IL-6 and IL-10, anti-inflammatory cytokine, expression respectively. **d** Serum LPS level of ND- and HFD-fed rats at 12th week. **e** Serum LPS level of ND- and HFD-fed rats after receiving prebiotics, probiotics, or synbiotics. ND: 12-week-normal diet-fed rats; HFD: 12-week high fat-fed rats; V: rats receiving PBS as vehicle; PE: rats receiving prebiotics; PO: rats receiving probiotics; C: rats receiving combination of prebiotics and probiotics as synbiotics (*N* = 6 of each group) *p < 0.05 in comparison with the ND-fed rats; †*p* < 0.05 in comparison with the HFD-fed rats receiving vehicle

Pro-inflammatory cytokine interleukin (IL)-1 and IL-6 mRNA expression levels were significantly increased in the colon of rats fed with a HFD compared to rats fed with a ND, whereas the immunosuppressive cytokine IL-10 mRNA level was not altered (Fig. 2a–c). Diet-induced obese rats also developed the metabolic endotoxemia, the increased LPS in their sera (Fig. 2d), which was ameliorated by consumption of prebiotic XOS, probiotic *L. paracasei* HIIO1, or the synbiotics (Fig. 2e). Collectively, chronic HFD consumption resulted in both local (colon) and systemic (metabolic endotoxemia) inflammation, and consumption of prebiotic XOS, probiotic *L. paracasei* HIIO1, or the synbiotics could significantly reduce these low-grade inflammations. In this study, the pro-inflammatory mRNA levels of IL-1β and IL-6 from the whole brain tissues were not different among groups (Table 2). However, the two hippocampi in each animal were sufficient only for protocol of dendritic spine, hippocampal ROS production, and Western blot analysis; therefore, we did not have enough hippocampal tissues for cytokine analysis.

Our findings demonstrated that HFD-induced gut dysbiosis, as indicated by an increased F/B ratio in HFD-fed rats treated with vehicle (0.479 ± 0.174), compared to that of ND-fed rats treated with vehicle (0.159 ± 0.235, *p* < 0.05). The F/B ratio of HFD-fed rats treated with prebiotic XOS (0.089 ± 0.312), probiotic *L. paracasei HII01* (0.167 ± 0.522), or synbiotics (0.160 ± 0.188) was equally reduced when compared with the F/B ratio of HFD-fed rats treated with vehicle (0.479 ± 0.174, *p* < 0.05). These findings indicated that long-term HFD consumption caused gut dysbiosis, and the supplement with prebiotics, probiotics, and synbiotics could attenuate gut dysbiosis, as indicated by decreased F/B ratio.

### Long-term HFD consumption caused peripheral insulin resistance and dyslipidemia, and treatments attenuated these metabolic disturbances

After 12 weeks of HFD consumption, the body weight, plasma insulin level, and HOMA index of HFD-fed rats increased significantly when compared to ND-fed rats without alteration of the plasma glucose level (Table 1). Moreover, rats fed with a HFD had significantly increased plasma total cholesterol and LDL cholesterol when compared to the ND-fed rats (Table 1). Interestingly, 12 weeks of prebiotic XOS, probiotic *L. paracasei* HIIO1 or the synbiotics supplements had significantly decreased plasma insulin level, HOMA index, area under the curve of the oral glucose tolerance test (AUCg), plasma total cholesterol level, and LDL cholesterol level when compared to the HFD-fed rats receiving the vehicle (Table 2). These findings suggested that long-term HFD consumption caused peripheral insulin resistance as indicated by hyperinsulinemia with euglycemia and increased HOMA index as well as dyslipidemia, which were improved by all treatments.

**Table 1:** The metabolic parameters at baseline and after 12 weeks of either ND or HFD consumption

Metabolic parameters	Baseline	ND	HFD
Body weight (g)	225 ± 2	459 ± 6*	540 ± 9*†
Food intake (g/day)	21 ± 0.5	21 ± 0.2	24 ± 0.2*†
Plasma glucose (mg/dl)	132.6 ± 6	137.7 ± 4	142.2 ± 5
Plasma insulin (ng/ml)	2.3 ± 0.3	4.6 ± 0.4*	6.0 ± 0.5*†
HOMA index	22.4 ± 5	41.7 ± 4*	63.4 ± 7*†
Plasma total cholesterol (mg/dl)	74.7 ± 2	72.5 ± 3	89.4 ± 3*†
Plasma total triglyceride (mg/dl)	61.1 ± 5	65.2 ± 3	66.1 ± 5
LDL cholesterol (mg/dl)	21.5 ± 3	21.9 ± 3	34.1 ± 3*†

*P < 0.05 in comparison with baseline group

†*P* < 0.05 in comparison with ND group

**Table 2:** The metabolic parameters after 12 weeks of vehicle, prebiotic, probiotic, or synbiotics administration in ND-fed rats and HFD-fed rats

Metabolic parameters	ND				HFD			
	NDV	NDPE	NDPO	NDC	HFV	HFPE	HFPO	HFC
Body weight (g)	501 ± 9	495 ± 11	517 ± 14	510 ± 13	680 ± 24*	605 ± 30*†	689 ± 33*	600 ± 34*†
Food intake (g/day)	20 ± 0.5	19 ± 0.7	21 ± 0.9	21 ± 0.4	25 ± 0.4*	23 ± 0.7*	25 ± 0.5*	24 ± 0.5*
Visceral fat (g)	25 ± 2	27 ± 3	29 ± 3	33 ± 2	63 ± 3*	43 ± 5*†	65 ± 3*	48 ± 5*†
Plasma glucose (mg/d)	132.3 ± 7	140.5 ± 8	137.5 ± 8	141.6 ± 12	139.2 ± 9	142.1 ± 6	137.8 ± 4	132.1 ± 14
Plasma insulin (ng/ml)	4.8 ± 0.8	5.5 ± 1	5.4 ± 0.8	4.0 ± 1	7.8 ± 0.5*	5.5 ± 0.5†	5.2 ± 1†	5.0 ± 1†
HOMA index	40.3 ± 10	50.0 ± 14	55.8 ± 11	50.9 ± 12	94.6 ± 12*	55.6 ± 6†	60.5 ± 8†	39.8 ± 5†
Plasma glucose AUC (AUCg) (mg/dl × min × 10^4^)	2.1 ± 0.1	2.0 ± 0.1	2.2 ± 1	2.3 ± 0.1	2.9 ± 0.1*	2.1 ± 0.1†	2.4 ± 0.1†	2.2 ± 0.1†
Plasma total cholesterol (mg/dl)	74.4 ± 4	68.7 ± 4	65.9 ± 5	58.6 ± 5	111.1 ± 8*	73.9 ± 3†	78.8 ± 4†	75.2 ± 6†
Plasma total triglyceride (mg/dl)	78.3 ± 13	71.5 ± 6	68.7 ± 8	77.7 ± 7	84.9 ± 10	73.3 ± 6	78.3 ± 4	74.5 ± 4
Plasma LDL cholesterol (mg/dl)	24.2 ± 5	22.3 ± 2	23.5 ± 6	22.7 ± 6	65.7 ± 10*	33.1 ± 4†	35.3 ± 5†	27.1 ± 5†
Serum MDA (μmol/dl)	3.72 ± 0.2	3.92 ± 0.1	3.91 ± 0.1	3.64 ± 0.2	5.76 ± 0.5*	3.31 ± 0.2†	3.23 ± 0.3†	3.16 ± 0.2†
Brain MDA (μmol/mg protein)	7.59 ± 1.8	8.10 ± 1.9	6.58 ± 1.6	2.01 ± 1.0*	15.0 ± 2.1*	8.23 ± 1.9†	5.59 ± 1.1†	1.84 ± 0.7*†
Brain IL-1β (fold change/gapdh)	1.39 ± 0.5	0.45 ± 0.1	2.03 ± 0.1	1.82 ± 0.4	0.35 ± 0.1	0.71 ± 0.3	1.08 ± 0.6	0.51 ± 0.2
Brain IL-6 (fold change/gapdh)	1.27 ± 0.5	1.30 ± 0.8	10.68 ± 5.4	7.54 ± 1.2	1.10 ± 0.4	1.29 ± 0.1	3.57 ± 2.7	0.80 ± 0.2

*P < 0.05 in comparison with the NDV group

†*P* < 0.05 in comparison with the HFV group

Before treatment, we found a negative correlation between time in target quadrant of probe test with the metabolic parameters including body weight (*r* = − 0.689, *p* < 0.01), insulin (*r* = − 0.658, *p* < 0.01), HOMA index (*r* = − 0.756, *p* < 0.01), plasma total cholesterol (*r* = − 0.724, *p* < 0.01), and serum LPS level (*r* = − 0.877, *p* < 0.01). After treatment with prebiotic, probiotic, or synbiotics, we found the negative correlations between time in target quadrant of probe test with the metabolic parameters and inflammatory markers including body weight (*r* = − 0.387, *p* < 0.01), plasma total cholesterol (*r* = − 0.388, *p* < 0.01), LDL cholesterol (*r* = − 0.492, *p* < 0.01), colon IL-6 mRNA expression (*r* = − 0.355, *p* < 0.01), fat mass (*r* = − 0.333, *p* < 0.01), serum LPS level (*r* = − 0.312, *p* < 0.05), and brain LPS level (*r* = − 0.466, *p* < 0.01). Taken together, these findings added the potential mechanism regarding the protective effects of prebiotic, probiotic, or synbiotics on cognitive dysfunction in obese-insulin resistant rat that could occur possibly through the modulation of LDL cholesterol level, fat mass, and serum and brain LPS level as well as the level of colon IL-6 mRNA expression.

### Prebiotic XOS, probiotic *L. paracasei* HIIO1, or the synbiotics restored hippocampal plasticity impaired by long-term HFD consumption

**Fig. 3:**
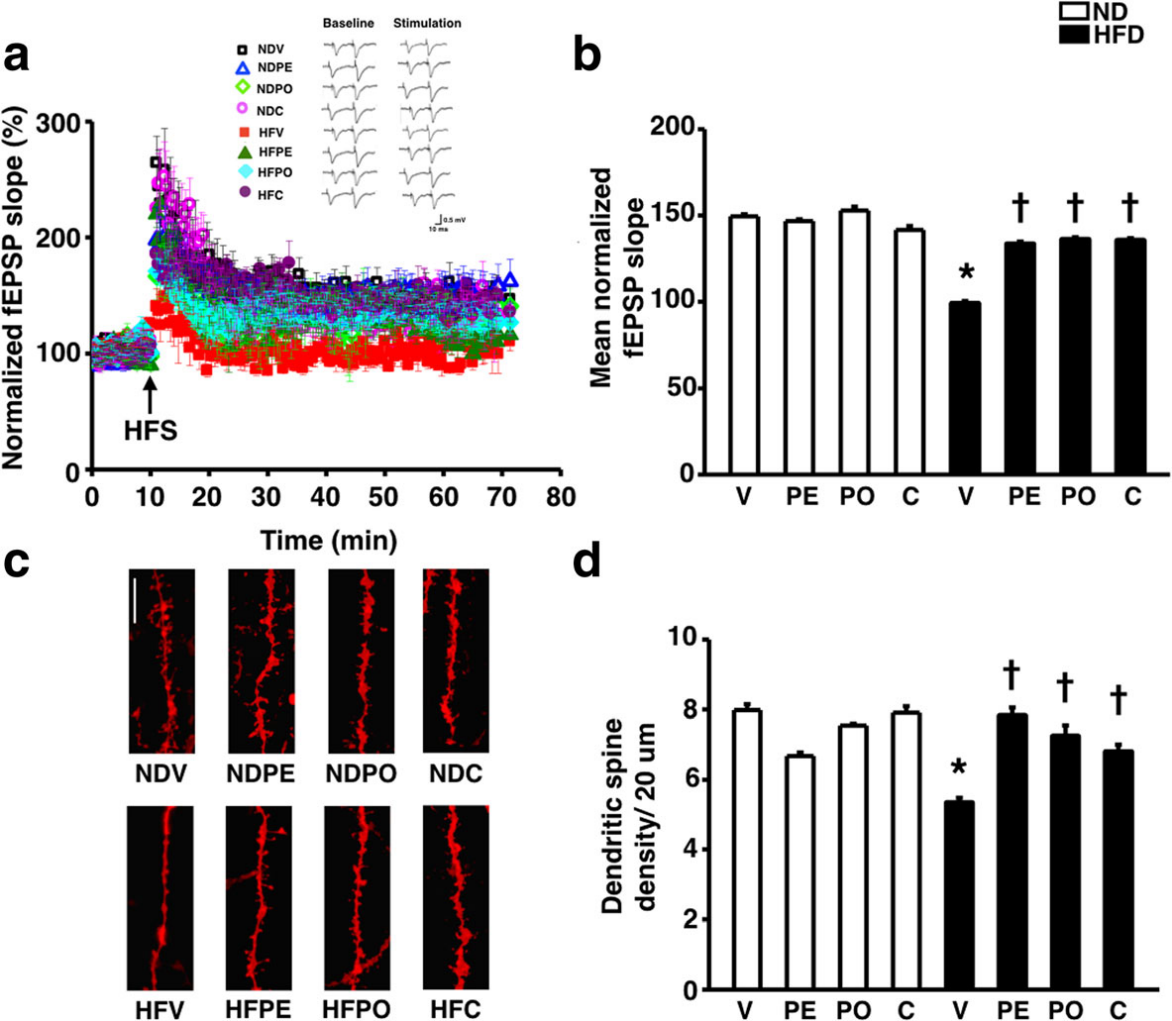
Effects of prebiotics, probiotics, or synbiotics on hippocampal plasticity. **a** Percentage normalized fEPSP slope of electrical-induced LTP by extracellular recording. **b** Mean fEPSP slope from 50 to 60 mins of electrical-induced LTP. **c** Representative images of Dil immunofluorescent under confocal microscopy (bar = 5 μm). **d** Mean dendritic spine density. ND: 24-week-normal diet-fed rats; HFD: 24-week high fat-fed rats; V: rats receiving PBS as vehicle; PE: rats receiving prebiotics; PO: rats receiving probiotics; C: rats receiving combination of prebiotics and probiotics as synbiotics (*N* = 6 of each group) *p < 0.05 in comparison with the ND-fed rats; †*p* < 0.05 in comparison with the HFD-fed rats receiving vehicle

To determine hippocampal plasticity, the fEPSP slope of LTP was measured from CA1 area of hippocampal slices. LTP is a marker of hippocampal synaptic plasticity. HFD-fed rats treated with the vehicle showed impaired hippocampal plasticity indicated by a significantly decreased mean fEPSP slopes compared to ND-fed rats, whereas all treatments effectively normalized the fEPSP slopes (*n* = 2–3 independent slices/animal, *n* = 6 animals/group; (Fig. 3a–b)) in these HFD rats. In addition, dendritic spine density was also significantly decreased in HFD rats, which was restored in HFD-fed rats treated with prebiotic XOS, probiotic *L. paracasei* HIIO1, or synbiotics (Fig. 3c–d). Taken together, long-term HFD consumption demonstrated hippocampal dysplasticity as indicated by impaired LTP and decreased dendritic spine density, and all treatments reversed these impairments.

### Prebiotic XOS, probiotic *L. paracasei* HIIO1, or the synbiotics improved brain mitochondrial dysfunction, hippocampal oxidative stress, and hippocampal apoptosis

**Fig. 4:**
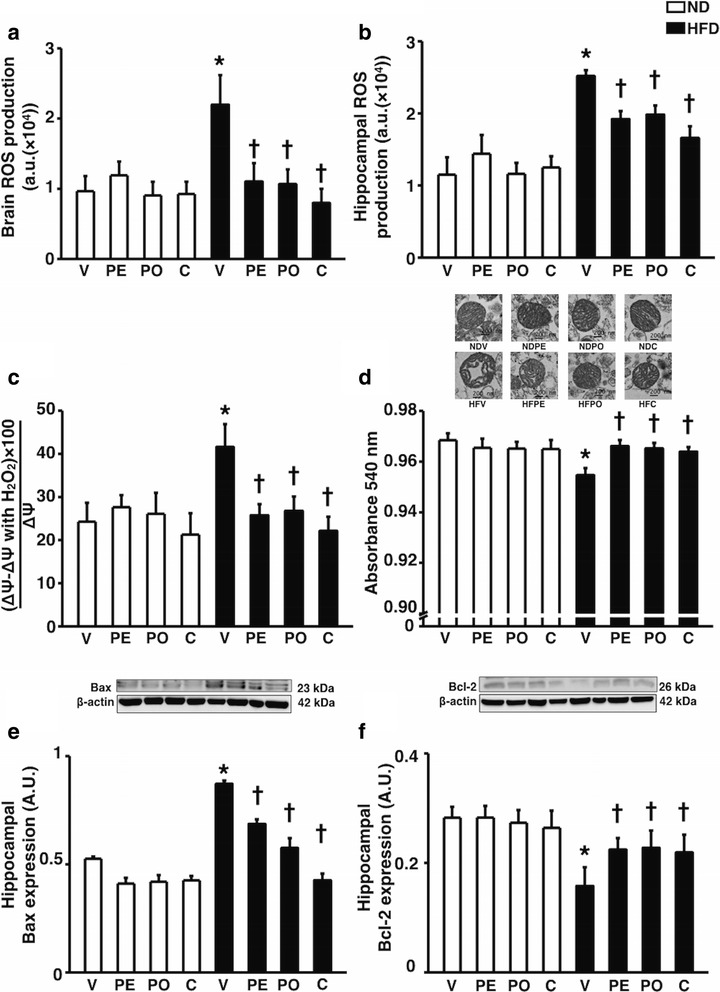
Effects of prebiotics, probiotics, or synbiotics on brain mitochondrial function, hippocampal oxidative stress, and hippocampal apoptosis. **a** Whole brain isolated mitochondrial ROS production. **b** Hippocampal ROS production. **c** Percent change of whole brain isolated mitochondrial depolarization when incubated with hydrogen peroxide. **d** Upper panel: representative images of brain mitochondrial morphology. Lower panel: whole brain isolated mitochondrial absorbance value. **e** Upper panel: representative immunoblotting images of Bax relative to actin expression. Lower panel: the expression of hippocampal Bax protein relative to actin. **f** Upper panel: representative immunoblotting images of Bcl-2 relative to actin expression. Lower panel: the expression of hippocampal Bcl-2 protein relative to actin. ND: 24-week-normal diet-fed rats; HFD: 24-week high fat-fed rats; V: rats receiving PBS as vehicle; PE: rats receiving prebiotics; PO: rats receiving probiotics; C: rats receiving combination of prebiotics and probiotics as synbiotics (*N* = 6 of each group) *p < 0.05 in comparison with the ND-fed rats; †*p* < 0.05 in comparison with the HFD-fed rats receiving vehicle

To determine brain mitochondrial function, the whole brain and hippocampus ROS production, brain mitochondrial depolarization, and brain mitochondrial swelling were measured. HFD-fed rats treated with the vehicle had increased brain and hippocampus ROS production (Fig. 4a–b), brain mitochondrial depolarization (Fig. 4c) as well as decreased brain mitochondrial absorbance indicating brain mitochondrial swelling (Fig. 4d). These impairments were attenuated by all treatments. In addition, to determine hippocampal apoptosis, the expression of apoptotic and anti-apoptotic proteins including bax and bcl-2 was determined. The increase of bax expression and decrease of bcl-2 expression found in HFD-fed rats treated with the vehicle was improved in HFD-fed rats receiving prebiotic XOS, probiotic *L. paracasei* HIIO1, or the synbiotics (Fig. 4e–f). These findings demonstrated that all treatments ameliorated brain mitochondrial dysfunction, decreased hippocampal oxidative stress levels, and exerted anti-apoptotic effects.

### Microglial activation was attenuated by prebiotic XOS, probiotic *L. paracasei* HIIO1, or the synbiotics in obese-insulin resistant rats

**Fig. 5:**
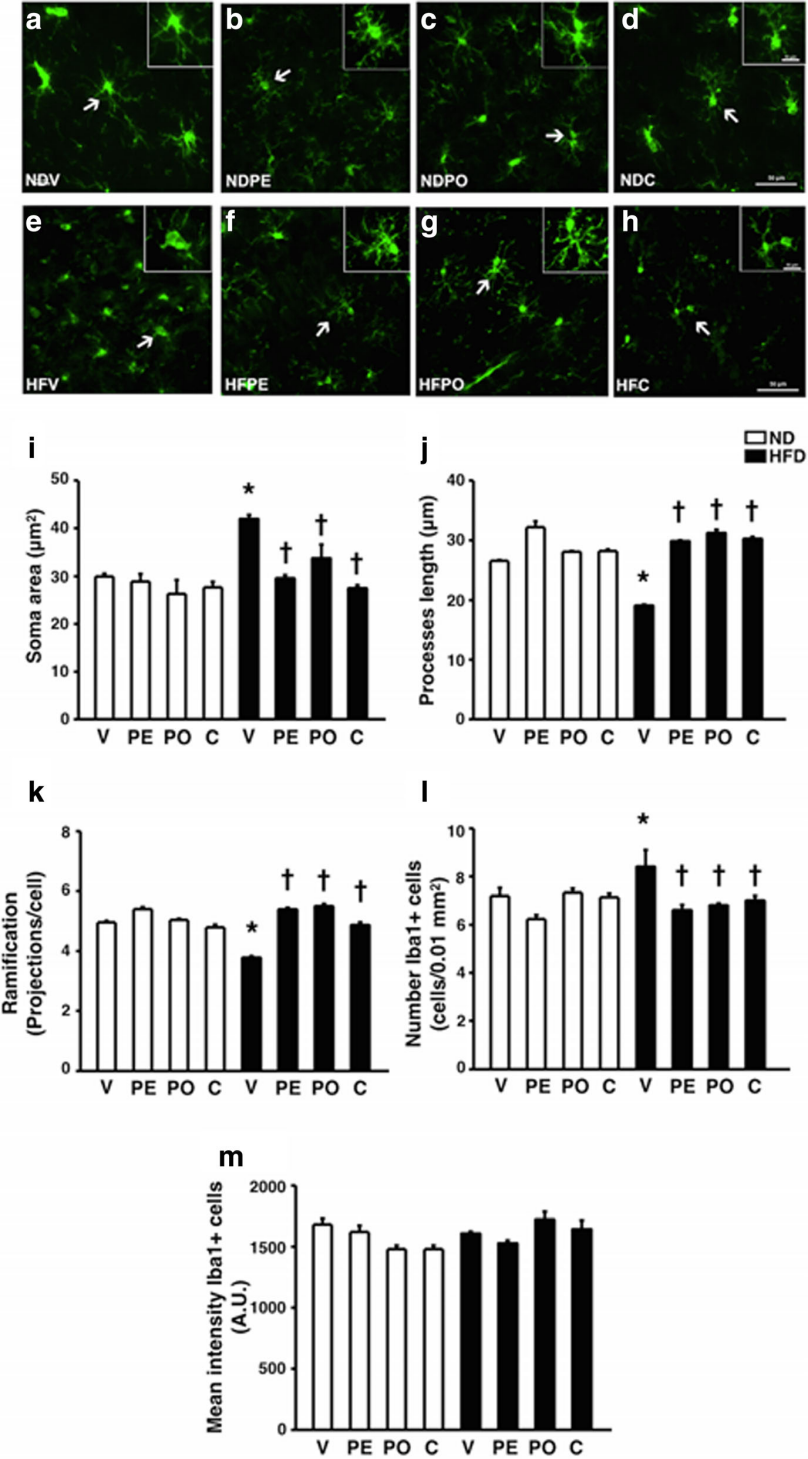
Effects of prebiotics, probiotics, or synbiotics on brain microglia morphology. **a**-**h** Representative images of Iba-1 immunofluorescent under confocal microscopy at CA1 of the hippocampus (bar = 50 μm). **i** Soma area of Iba-1 positive cell. **j** Processes length of Iba-1 positive cell. **k** The ramification of Iba-1 positive cell. **l** Number Iba-1 positive cell. **m** Mean fluorescent intensity of Iba-1 positive cell. ND: 24-week-normal diet-fed rats; HFD: 24-week high fat-fed rats; V: rats receiving PBS as vehicle; PE: rats receiving prebiotics; PO: rats receiving probiotics; C: rats receiving combination of prebiotics and probiotics as synbiotics (3 microglial calls/slice, 3 slices/animal and 6 animals/ group) *p < 0.05 in comparison with the ND-fed rats; †*p* < 0.05 in comparison with the HFD-fed rats receiving vehicle

To determine microglia morphology phenotype, soma size, and processes length, ramification number Iba-1 positive cell and mean fluorescent intensity were measured. Three microglial cells per brain slice, three brain slices per animal, and six animals per group were measured from the CA1 region of the hippocampus. The microglial morphology of Iba-1 immunofluorescent under confocal microscopy at CA1 of the hippocampus were demonstrated (Fig. 5a–h). There were no significant differences in all microglial morphology parameters among the ND-fed groups (Fig. 5a–d). Microglia from HFD-fed rats had amoeboid phenotype (Fig. 5e) as indicated by the significantly increased soma size (Fig. 5i), decreased process length (Fig. 5j), increased major projection and increased Iba-1 positive cell when compared to ND-fed rats (Fig. 5k–m). Prebiotic XOS, probiotic *L. paracasei* HIIO1, or the synbiotics preserved all microglial morphology parameters (Fig. 5i–m). Collectively, HFD consumption led to microglial morphology changes which were attenuated in all treatments.

### Cognitive dysfunction induced by long-term HFD consumption was ameliorated in prebiotic XOS, probiotic *L. paracasei* HIIO1, or the synbiotics consumption

**Fig. 6:**
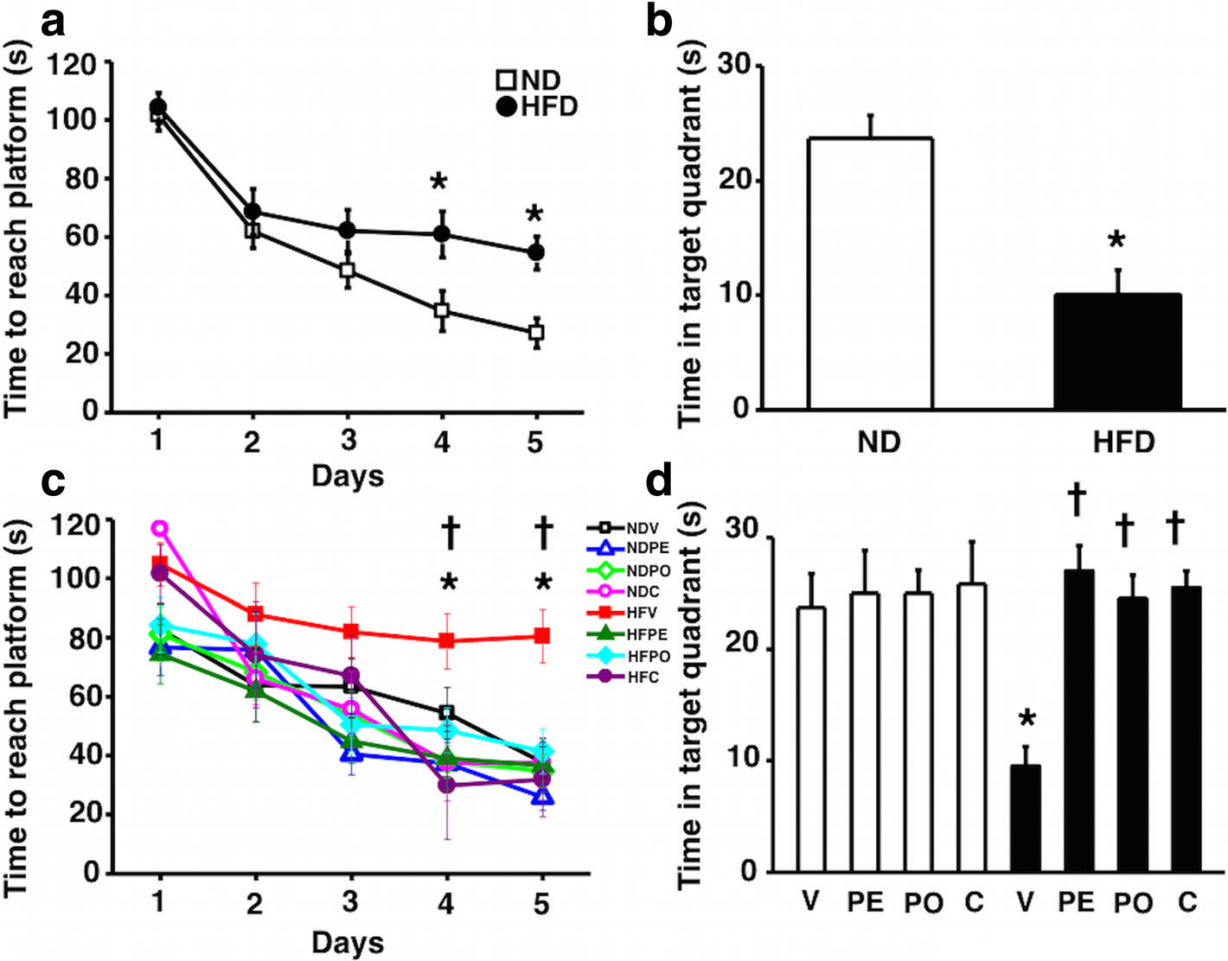
Effects of prebiotics, probiotics, or synbiotics on cognitive function. **a** Time to reach the platform in acquisition test of Morris Water maze test of ND- and HFD-fed rats at 12th week. **b** Mean time spent in target quadrant of ND- and HFD-fed rats at 12th week. **c** Time to reach the platform in acquisition test of Morris Water maze test after receiving prebiotics, probiotics, or synbiotics. **d** Mean time spent in target quadrant after receiving prebiotics, probiotics, or synbiotics. ND: 24-week-normal diet-fed rats; HFD: 24-week high fat-fed rats; V: rats receiving PBS as vehicle; PE: rats receiving prebiotics; PO: rats receiving probiotics; C: rats receiving combination of prebiotics and probiotics as synbiotics (*N* = 6 of each group) *p < 0.05 in comparison with the ND-fed rats; †*p* < 0.05 in comparison with the HFD-fed rats receiving vehicle

Cognitive function was determined by Morris water maze test. Twelve weeks of HFD consumption caused memory impairment as indicated by the increased time taken to reach the platform (Fig. 6a) and decreased time spent in the target quadrant in these rats, compared to 12-week ND-fed rats (Fig. 6b). After 12 weeks of receiving prebiotic XOS, probiotic *L. paracasei* HIIO1, or the synbiotics in HFD-fed rats, the time to reach the platform was significantly decreased when compared to the vehicle group during the acquisition test (Fig. 6c). In addition, the time spent in the target quadrant during the probe test in HFD-fed rats with prebiotic XOS, probiotic *L. paracasei* HIIO1, or the synbiotics was also significantly higher than that of the vehicle group (Fig. 6d). All of these findings suggested that all treatments effectively attenuate the impairment of learning and memory behaviors caused by long-term HFD consumption. The locomotor activity was determined by the open-field test. We found that long-term HFD consumption did not alter locomotor activity, indicating by distance (cm/10 min), when compared to ND-fed rats (2406 ± 560 cm vs. 2423 ± 690 cm for ND-fed rats and HFD-fed rats, respectively). Moreover, the locomotor activity of ND-fed rats and HFD-fed rats treated with prebiotic XOS, probiotics *L. HII01*, or synbiotics also was not significantly different when compared to ND-fed rats treated with vehicle (NDV 2368 ± 152 cm; NDPE 2658 ± 611 cm; NDPO 3038 ± 340 cm; NDC 2219 ± 444 cm; HFV 2498 ± 707 cm; HFPE 2542 ±646 cm; HFPO 2808 ± 686 cm; and HFC 3135 ± 1389 cm). These findings also indicated that the cognitive impairment during the Morris water maze test did not involve the motor function.

## Discussion

The major findings of the present study are as follows. The obesity caused by long-term HFD consumption had (1) low-grade inflammation found in both local (gut) and systemic (serum) sites, leading to low-grade systemic inflammation and the development of peripheral insulin resistance; (2) hippocampal dysplasticity; (3) brain mitochondrial dysfunction; and (4) cognitive decline. These impairments are possibly mediated through the induction of gut inflammation, brain and hippocampal oxidative stress, brain inflammation, hippocampal apoptosis, the reduction of dendritic spine density, and microglial dysfunction. Daily consumption of prebiotic XOS, probiotic *L. paracasei* HIIO1, or the synbiotics for 12 weeks improved the brain function in these obese rats by attenuating gut and systemic inflammations, decreasing brain and hippocampal oxidative stress, increasing dendritic spine density, ameliorating microglial activation, and improving hippocampal dysplasticity and brain mitochondrial dysfunction, leading to restored cognitive function.

Previous studies demonstrated that long-term HFD consumption is known to lead to gut dysbiosis by enhancing the growth of *Proteobacteria*, which is mainly composed of Gram-negative LPS containing bacteria, in the gut in the gut content [18, 19] and impaired the gut barrier integrity by inhibition of tight junction proteins [53]. This “leaky gut” found in the obese mice allows the luminal LPS and LPS-containing bacteria translocated from gut lumen to activate the innate immune cells in gut lamina propria, thus triggering the inflammatory response [18, 54]. Consistent with those reports, long-term HFD consumption in this study caused gut inflammation and increased the LPS level, in which it is possible that that amount of *Proteobacteria* should be increased in our HFD-fed rats. These findings suggested that obesity induced by HFD consumption caused gut inflammation, leading to low-grade systemic inflammation and the development of a peripheral insulin resistance. These undesirable effects were attenuated by consumption of prebiotics, probiotics, or synbiotics. In this study, the pro-inflammatory mRNA levels of IL-1β and IL-6 in the brain were not different among groups. Since this was done in the whole brain tissues, future studies are needed to investigate whether the pro-inflammatory cytokines in the hippocampal tissues would be different between the treatment groups and the control groups.

Although we found that only prebiotic XOS and synbiotics, not probiotic *L. paracasei* HIIO1, attenuated adiposity, which was the major source of pro-inflammatory cytokines, by decreasing the body weight and visceral fat, insulin resistance and dyslipidemia were still improved in all treatments. Currently, the beneficial role of probiotics on metabolic syndrome is still debated, at least one part was due to the strain-specific effect. For example, oral supplement of *Lactobacillus acidophilus*, *Lactobacillus ingluviei*, and *Lactobacillus fermentum* can cause weight gain [55], whereas *Lactobacillus gasseri* and *Lactobacillus rhamnosus* promoted weight loss [56]. Collectively, prebiotic XOS and synbiotics had beneficial effects to metabolic disturbance through systemic inflammation stemming from gut dysbiosis and adiposity, whereas probiotic *L. paracasei* HIIO1 had beneficial effects through systemic inflammation stemming only from gut dysbiosis. Our findings on probiotic supplement support this hypothesis.

In addition, blood-brain barrier (BBB) permeability was increased in a model of obesity-induced by HFD [57] and also aggravated cognitive deficit by increasing the exposure of the brain to various cytokines, including LPS, IL-1β, IL-6, and tumor necrosis factor alpha (TNFα) [58]. These undesirable effects were diminished in rats receiving prebiotic XOS, probiotic *L. paracasei* HIIO1, or synbiotics and restored cognitive function, possibly modulated through anti-oxidative and anti-inflammatory effects. Growing evidence demonstrates that the supplementary XOS decreased oxidative status in white sea bream juvenile [59] and suppressed pro-inflammatory cytokines including IFNγ and IL-1β [60]. Probiotics are also known to exert an anti-inflammatory effect since it has been shown previously that *Lactobacillus helveticus* decreased inflammatory markers including nitric oxide synthase (NOS), prostaglandin E2 (PGE2), and IL-1β in the brain [25]. In addition, synbiotics, the combination of XOS and *Lactobacillus plantarum*, had greater antioxidant activity than single therapy, indicating that prebiotics, probiotics, or synbiotics could effectively decrease oxidative stress and inflammation not only in the gut and circulation, but also in the brain [61]. Taken together, prebiotic XOS, probiotic *L. paracasei* HII01, or synbiotics (the combination of XOS and *L. paracasei* HII01) exerted an anti-oxidative effect and anti-inflammatory effect, leading to restored cognitive function impaired by HFD. Previous studies also demonstrated that obesity-induced cell apoptosis by increased Bax level, decreased Bcl-2 level, and impaired brain mitochondrial function, which were also seen in the present study [48, 62]. Interestingly, we found that prebiotic XOS, probiotic *L. paracasei* HIIO1, or the synbiotics attenuated brain mitochondrial dysfunction, hippocampal ROS production, and hippocampal apoptosis.

Growing evidence has demonstrated the crucial roles of microglia on cognitive dysfunction in neurodegenerative disorders including excessive synaptic pruning of the brain with Alzheimer’s disease [29, 30] and robust brain inflammation in obesity [31–33]. Previous studies demonstrated that HFD consumption increased activated microglia, leading to hippocampal dysplasticity including impairment of LTP, decreased dendritic spine density, as well as decreased synaptic protein such as postsynaptic density protein 95 (PSD-95), synaptophysin, and spinophilin, resulting in cognitive dysfunction [31, 33].

Although microglia played a crucial role in cognitive function and prebiotics, probiotics or synbiotics have been shown to exert protective effects on cognition [26, 30]; the link between gut microbiota modulating cognitive function through microglia has never been tested. In the present study, we demonstrated for the first time that long-term consumption of prebiotic XOS, probiotic *L. paracasei* HIIO1, or the synbiotics ameliorated microglial activation and restored cognitive function in diet-induced obese rats. There are three possible mechanistic explanations for the beneficial effects of the interventions we used in this study with prebiotics, probiotics, and synbiotics on brain cognitive function. First, prebiotics, probiotics, and synbiotics can mediate their effects through vagus nerve activation. It has been shown that consumption of *Bifidobacterium longum* exerted a vagal pathway-dependent anxiolytic effect in a chemically induced colitis mouse model [63]. Second, prebiotics, probiotics, and synbiotics can attenuate microglial activation which occurs in response to metabolite profiles in diet-induced obesity [18, 19]. Third, the gut microbiota can increase the production of short-chain fatty acids (SCFAs) such as acetate, butyrate, and proprionate, which has been shown to be beneficial in metabolic syndrome [64, 65]. However, the beneficial effects of prebiotics, probiotics, or synbiotics are strain-specific. Further research is needed into the mechanisms behind the role of microglia in cognition and the signaling pathways involved in neuroglia communication.

Surprisingly, the synbiotics did not have the synergistic effect of the attenuation of inflammation, hippocampal oxidative stress, hippocampal apoptosis, mitochondrial dysfunction as well as microglial dysfunction in rats with an obese-insulin resistant condition. These findings suggest that the prebiotic XOS might not effectively promote the probiotic functions of *L. paracasei HII01* in vivo. Moreover, these observations suggest that inconsistent reports regarding the probiotic effect in the treatment of metabolic syndrome could be due to a strain-specific effect of this probiotic in a combination with a specific prebiotic fiber. This possibility has been supported by previous studies which demonstrated that XOS could not facilitate the growth of *Lactobacillus paracasei* [66, 67].

## Conclusion

The present study showed that obese-insulin resistant condition, induced by prolonged HFD consumption, causes gut and systemic inflammation, peripheral insulin resistance, hippocampal dysplasticity, hippocampal oxidative stress, brain mitochondrial dysfunction, hippocampal apoptosis, and microglial morphological changes, resulting in cognitive decline. Moreover, this is the first report to show the possible link between gut microbiota modification by prebiotics, probiotics, or synbiotics supplement and the improvement of cognitive function in obese-insulin resistant rats. These neuroprotective effects may possibly be mediated through the attenuation of inflammation, hippocampal oxidative stress, hippocampal apoptosis, mitochondrial dysfunction as well as microglial dysfunction.

## Abbreviations

aCSF: Artificial cerebrospinal fluid
AUG: Areas under the curve
cDNA: Complementary DNA
cfu: Colony forming unit
fEPSP: Field excitatory postsynaptic potentials
HFD: High-fat diet
HFS: High-frequency stimulation
HOMA: Homeostasis Model Assessment
HPLC: High-performance liquid chromatography
IL-1: Interleukin 1
IL-6: Interleukin 6
LAL: Limulus amoebocyte lysate
LPS: Lipopolysaccharide
LTP: Long-term potentiation
MDA: Malondialdehyde
ND: Normal diet
OGTT: Oral glucose tolerance test
ROS: Reactive oxygen species
XOS: Xylooligosaccharide
